# Brainstem Involvement as a Cause of Central Sleep Apnea: Pattern of Microstructural Cerebral Damage in Patients with Cerebral Microangiopathy

**DOI:** 10.1371/journal.pone.0060304

**Published:** 2013-04-23

**Authors:** Thomas Duning, Michael Deppe, Eva Brand, Jörg Stypmann, Charlotte Becht, Anna Heidbreder, Peter Young

**Affiliations:** 1 Department of Neurology, University Hospital of Muenster, Muenster, Germany; 2 Internal Medicine D, Department of Nephrology, Hypertension and Rheumatology, University Hospital Muenster, Muenster, Germany; 3 Department of Cardiovascular Medicine, Division of Cardiology, University Hospital Muenster, Muenster, Germany; 4 Department of Neurology, Section for Sleepmedicine, University Hospital Muenster, Muenster, Germany; Julius-Maximilians-Universität Würzburg, Germany

## Abstract

**Background:**

The exact underlying pathomechanism of central sleep apnea with Cheyne-Stokes respiration (CSA-CSR) is still unclear. Recent studies have demonstrated an association between cerebral white matter changes and CSA. A dysfunction of central respiratory control centers in the brainstem was suggested by some authors. Novel MR-imaging analysis tools now allow far more subtle assessment of microstructural cerebral changes. The aim of this study was to investigate whether and what severity of subtle structural cerebral changes could lead to CSA-CSR, and whether there is a specific pattern of neurodegenerative changes that cause CSR. Therefore, we examined patients with Fabry disease (FD), an inherited, lysosomal storage disease. White matter lesions are early and frequent findings in FD. Thus, FD can serve as a "model disease" of cerebral microangiopathy to study in more detail the impact of cerebral lesions on central sleep apnea.

**Patients and Methods:**

Genetically proven FD patients (n = 23) and age-matched healthy controls (n = 44) underwent a cardio-respiratory polysomnography and brain MRI at 3.0 Tesla. We applied different MR-imaging techniques, ranging from semiquantitative measurement of white matter lesion (WML) volumes and automated calculation of brain tissue volumes to VBM of gray matter and voxel-based diffusion tensor imaging (DTI) analysis.

**Results:**

In 5 of 23 Fabry patients (22%) CSA-CSR was detected. Voxel-based DTI analysis revealed widespread structural changes in FD patients when compared to the healthy controls. When calculated as a separate group, DTI changes of CSA-CSR patients were most prominent in the brainstem. Voxel-based regression analysis revealed a significant association between CSR severity and microstructural DTI changes within the brainstem.

**Conclusion:**

Subtle microstructural changes in the brainstem might be a neuroanatomical correlate of CSA-CSR in patients at risk of WML. DTI is more sensitive and specific than conventional structural MRI and other advanced MR analyses tools in demonstrating these abnormalities.

## Introduction

Central sleep apnea with Cheyne-Stokes respiration (CSA-CSR) is a form of sleep-disordered breathing. Central sleep apneas (CSA) and hypopneas arise from complete or partial reductions in central neural outflow to the respiratory muscles during sleep. They are characterized by a cessation or decrease of ventilatory effort for at least 10 seconds during sleep and are usually associated with oxygen desaturation [1]. CSA-CSR is characterized by central apneas and alternating hyperpneas with regular “crescendo-decrescendo”/waxing-waning fluctuations in respiratory rate and tidal volume [2]. CSA-CSR is a relatively rare observation in sleep laboratory patients, as compared to obstructive sleep apnea, but is considered a rather serious form of sleep-disordered breathing. The presence of CSA-CSR is associated with increased mortality and morbidity and has a significant impact on quality of life [3].

Common predisposing factors for CSA-CSR are enhanced central and peripheral chemosensitivity [4], [5], hypocapnia, and the presence of left ventricular systolic dysfunction [6]. CSA-CSR is seen in 40–60% of patients with heart failure (HF), but was also frequently found in patients with normal heart function [7]. The exact underlying pathomechanism of CSA-CSR is still unclear. It is unknown why some patients with heart failure are prone to develop CSA-CSR and others are not. CSA-CSR was also commonly reported in patients with isolated brainstem lesions or supratentorial strokes and normal heart function [5], [8]–[13]. Recent studies have demonstrated an association between cerebral white matter (WM) changes and central sleep-disordered breathing [14], [15]. It was suggested that the increased sensitivity to CO_2_ results from bilateral supramedullary brain lesions that disinhibit neural stimulatory input into the brainstem respiratory center (neurogenic CO_2_ hypersensitivity) [5]. In fact, the first case report of CSA-CSR described by Cheyne in 1818 concerned a male who suffered from both congestive heart failure and a stroke [16]. Subsequently, the question arose as to whether CSA-CSR was primarily related to cardiac or neurologic dysfunction [5], in particular to dysfunction of respiratory control centers in the brainstem [17]. However, neuroimaging (particularly conventional magnetic resonance imaging (MRI)) almost always fails to identify primary or secondary structural changes of the brainstem in patients with CSA-CSR.

Recent technological advances now allow far more subtle assessment of structural brain integrity. Diffusion Tensor Imaging (DTI) is an MR-imaging technique that evaluates WM integrity by measuring the spatial directionality of water diffusion, which is quantified by fractional anisotropy (FA) [18]. Axonal degeneration or other microstructural alterations of WM tracts reduce FA below normal values of unaffected individuals [19]. Thus, DTI allows detecting even subtle pathological changes of fiber integrity [20], [21]. Recent studies found that changes in FA have functional relevance as they have been shown to correlate with clinical symptoms and histopathological changes in early stages of neurodegeneration [22]–[25].

Fabry disease (FD; Online mendelian inheritance in man (OMIM) #301500) is an X-linked lysosomal storage disorder characterized by a deficient activity of the enzyme α-galactosidase A (GLA; OMIM #300644), caused by mutations in GLA coding region. The resulting accumulation of globotriaosylceramide leads to a multisystem disease [26]. The cellular dysfunction causes tissue remodelling and fibrosis, but mainly vasculopathic changes with ischemia, and ultimately leads to severe end-organ damage. Compared to the general population, the life span of untreated male and female Fabry patients is reduced by about 20 and 15 years, respectively [26]–[28]. The organ manifestations with the greatest impact in terms of reduced life span are the kidney and the brain. Other typical organ manifestations are those affecting the eyes, skin, heart (left ventricular hypertrophy and myocardial fibrosis during later stages) and peripheral nervous system. The reported incidence of FD varies substantially, ranging from 1∶40.000 to 1∶117.000, suggesting that the true incidence is much higher [28]. Prospective studies in patients with cryptogenic ischemic stroke, for instance, revealed a mutation in the GLA gene in 2 to 4% of the study population [29]. From the neurologist's point of view, FD is unique among lysosomal storage disorders. Although the clinical presentation of FD is variable, neurological symptoms are the most common clinical feature and in most patients also the initial manifestations of FD [30]–[32]. Fabry patients are typically seen by neurologists for cerebral ischemia, leukoencephalopathy of unknown cause, or neuropathic pain due to small-fiber neuropathy. Especially the marked cerebral vasculopathy with subsequent white matter lesions (WMLs) are relatively early and frequent findings in FD patients [32]. WML often are the first manifestation found in FD patients, and progressive WMLs have even been reported in children with FD [33]. Due to the young age of onset and the resulting significantly lower comorbidity, FD is a rather pure form of cerebral microangiopathy that can serve as a model disease of cerebral WM changes. Additionally, excessive daytime sleepiness is a common symptom in FD patients [34].

The objective of this study was to evaluate whether and to what extent subtle structural brain changes are associated with CSA-CSR in patients at risk of developing microangiopathic cerebral changes, and whether there is a specific pattern of microstructural cerebral changes that cause CSA-CSR. Therefore, we conducted cardiorespiratory polysomnography and a comprehensive clinical examination in 23 patients with genetically proven FD. We additionally applied different MR-imaging techniques, ranging from semiquantitative measurement of WM hyperintensities, automated calculation of brain parenchyma volumes, and voxel-based morphometry (VBM), to diffusion tensor imaging to detect even subtle neural changes.

## Methods

### Subjects

23 FD patients (12 males; mean age 46 years; range 29–61 years) from the center for FD in Münster, Germany, were recruited for the study. 44 healthy volunteers (23 males; mean age 46 years; range 29 to 59 years), all with normal neurological and physical examinations, were chosen to match the patients with regard to age and sex. FD patients underwent a standardized face-to-face medical interview, non-fasting blood sampling, physical and neurological examination by a trained study physician, electroneurography (ENG) of at least two peripheral nerves, a nocturnal cardio-respiratory polysomnography, and MRI of the brain at 3.0 Tesla. Left ventricular end-diastolic diameter (LVEDD), interventricular septal wall thickness (IVSD), and ejection fraction (EF) were determined by three-dimensional transthoracic echocardiography as recommended by the American Society of Echocardiography [35]. The definition of chronic kidney disease is based on the presence of kidney damage (ie, albuminuria/proteinuria) or decreased kidney function (ie, GFR <60 mL/min per 1·73 m^2^) for 3 months or more [36].

For enzymatic and molecular diagnosis of FD, standard methods were used as recommended [28]. All patients had been receiving enzyme replacement therapy (ERT) with agalsidase beta (1 mg/kg body weight intravenously every 2 weeks) for a mean of 4.8 years (range 0.9–7.9 years) at study enrollment. To determine disease severity, patients were classified according to the previously described Mainz Severity Score Index (MSSI). The MSSI is then divided into severity bands of mild (<20), moderate (20–40) and severe (>40) affliction, qualifying general, neurological, cardiovascular, and renal abnormalities [37]. Furthermore, sleepiness was measured using the Epworth Sleepiness Scale (ESS), a well-established tool for measuring daytime sleepiness in adults [38]. Patients were asked to rate the chances of dozing off or falling asleep in eight situations encountered in daily life on a scale of 0 to 3, with scores ranging from 0 to 24. Reliability of the instrument is 0.88, with validity correlated with the mean sleep latency scale at −0.30 (P<.001) [39], [40].

### Polysomnography

All FD patients underwent a nocturnal cardio-respiratory polysomnography (PSG) at the Center of Sleep Medicine at the University Hospital Muenster. Real-time monitoring of polysomnography was validated according to the definitions of the American Academy of Sleep Medicine (AASM) by certified sleep technicians and supervised by board-certified sleep specialists (PY and AH). Participants had their sleep measured by an in-lab 16-channel polysomnography (Nihon Kohdon). This method includes electroencephalogram, electrooculogram, electrocardiogram, snoring, chin and limb electromyelogram, chest/abdominal inductance respiratory belts, finger oximetry, body position and airflow monitoring with nasal cannula to record flow signals. Sleep records were manually scored in 30-sec epochs according to AASM standard criteria [2]. An apnea was defined as cessation of flow for ≥10 sec, and a hypopnea as reduction of ≥50% in flow amplitude lasting ≥10 sec. The respiratory event was scored as obstructive apnea (meeting the apnea criteria and associated with continued or increased respiratory effort throughout the entire period of absent airflow [41]) or as central apnea (meeting apnea criteria and associated with absent respiratory effort throughout the entire period of absent airflow). CSA-CSR was defined as follows: (1) at least 3 consecutive cycles of a cyclic crescendo-decrescendo change in the breathing amplitude and (2) central apnea-hypopnea index (AHI) ≥5 events/h or a cyclic crescendo-decrescendo change in breathing amplitude lasting at least 10 consecutive minutes. Total AHI, central AHI and obstructive oAHI were defined as number of respective events per hour of sleep. To quantify the severity of CSR, a CSR-ratio was calculated as duration of CSR episodes/total duration of sleep.

### MRI acquisition

Imaging data were obtained using a 3-Tesla system (Magnetom TIM Trio; Siemens, Erlangen, Germany). High resolution structural T1-and T2-weighted, and fluid attenuation inversion recovery (FLAIR) scans were acquired. MRI data acquisition was performed with the following parameters: T1: 3D magnetization-prepared rapid gradient echo (MPRAGE) sequence (repetition time (TR) 1900 ms, echo time (TE) 2.52 ms, NEX 1, flip angle 9 deg, matrix 256×256×160 over a FOV of 256×256×176 mm^3^, cubic voxel edge length of 1.0 mm); T2: TR = 4220, TE = 102 ms; FLAIR imaging: TR = 9000 ms, TE = 93 ms, TI = 2500 ms. For DTI, we employed echo planar imaging (EPI) with 20 diffusion directions (two b-factors, 0 s/mm^2^ and 1000 s/mm^2^, TR = 7 s/TE = 104 ms, voxel size: 1.80×1.80×3.6 mm, 2 averages) according to Jones and colleagues [42].

### Images analysis

Structural MRI analysis was performed by a single experienced observer blinded to clinical data. WML load was determined semiautomatically by outlining the peripheral borders of WML on axial FLAIR sequences. Lesions were marked and borders were set by local thresholding using a custom-tailored software based on Analyse-software (Brain Imaging Resource, Mayo Clinic, Rochester, MI, USA). By multiplying with the interslice distance, total volume of WML was established. Intra- and interobserver reliability was high with a weighted kappa of 0.98 and 0.93, respectively.

WMLs were additionally rated on a 3-point scale according to the well-established periventricular score of Fazekas [43]: 0 = no WML, 1 = punctate foci of WML, 2 = beginning confluence of foci of WMLs, and 3 =  large confluent areas of WMLs.

Global brain tissue volumes, normalized for subject head size, were calculated from T1-weighted images, using the cross-sectional version of the Structural Imaging Evaluation of Normalized Atrophy (SIENA) software (SIENAx) [44]. This method is an automated brain volume analysis tool that has been validated in healthy controls and patient data [45]. SIENAx first segments the image volume into brain and skull. The brain-only volume is then spatially normalized to the Montreal Neurological Institute (MNI) stereotactic space using an affine registration. The skull-only volume is used to obtain an appropriate scaling factor to limit normalization error due to atrophy. Then, tissue classification including partial volume estimation is performed, from which estimates of gray matter, WM and cerebrospinal fluid volumes are calculated. Finally, atrophy is assessed by calculating a brain parenchyma fraction as the ratio of the (normalized) brain parenchyma volume to the sum of the parenchyma and cerebrospinal fluid volume, i.e., as percentage of intracranial volume.

Patterns of gray matter atrophy were assessed using the automated and unbiased technique of voxel based morphometry (VBM). An optimized method of VBM was applied using both customized templates and prior probability maps, implemented using SPM5 software. The processing steps were performed as previously described [46]. Briefly, all images were normalized to a customized template and segmented by the unified segmentation procedure in SPM5 using the customized tissue probability maps into gray matter, WM and CSF, followed by the hidden Markov random field clean-up step. All images were modulated, and smoothed with a 12-mm full-width at half maximum smoothing kernel.

For DTI analyses, the echoplanar images (EPI) were spatially normalized to the Montréal Neurological Institute's (MNI) coordinate system after correction for eddy currents, following an optimized procedure on the basis of multiple image contrasts [47]. First, the diffusion tensor and FA field maps of all participants were calculated from spatially normalized images. In a second step, all FA images were normalized to an FA template image also corresponding to the MNI coordinate system. FA images were smoothed with an 8 mm isotropic Gaussian filter. These steps and the voxel-based analysis of FA were performed using the Münster neuroimaging analysis system *EVAL* [48], Matlab software (Mathworks Inc., Natick, MA) and Statistical Parametric Mapping (SPM5; Wellcome Department, London; http://www.fil.ion.ucl.ac.uk/spm/).

### Statistical analysis

Pearsońs correlation and Student t-test were used to assess associations of polysomnographic parameters with demographic data, clinical values (MSSI, ESS, cardiac parameters (LVEDD, IVSD, and EF), and imaging data (brain tissue volumes, WML volumes and - scores).

Based on the results of polysomnography, FD patients were divided into two groups: Patients with CSA-CSR and patients without CSA-CSR. Differences in clinical and imaging data (brain tissue volumes, WML volumes and Fazekas scores) between these groups were assessed using a two-sample t-test (P<0.05).

Voxel-wise differences in FA and gray matter values between all FD patients and healthy controls were statistically evaluated by analysis of covariance (ANCOVA), modeling age as a covariate to account for the age dependency of FA and gray matter. Statistical threshold for the correlation analysis was set at P<0.001, corrected for multiple comparison using the false discovery rate method (FDR-correction, minimum cluster size 50 voxels [49]), which is a standard approach in Statistical Parametric Mapping. In addition, voxel-wise FA and gray matter analyses of patients were performed as separate groups (with and without CSA-CSR), relative to the healthy controls, by using the same statistical parameters.

Furthermore, the linear regression tool of the SPM5 software was used to correlate maps of decreased FA and gray matter values with polysomnographic parameters (AHI, cAHI, oAHI, total and relative length of CSR episodes, mean SpO_2_, SpO_2_ nadir, SpO_2_<90%, sleep efficiency) in the group of FD patients with CSA-CSR (P<0.001, FDR-correction; minimum cluster size 50 voxels).

Statistical analyses outside of SPM5 were performed using SPSS 16.0.0. All data are given as means±SD. A two-sided P value<0.05 was considered significant, unless stated otherwise.

### Ethics

The study, conducted in strict accordance to the principles expressed in the Declaration of Helsinki, was approved the ethics committee of the Westphalian Medical Association and the faculty of medicine at the University of Münster, Germany (Ref.-number 2011-142-f-S). All participants in this study gave written informed consent.

Results

Average disease severity of the FD patients was mild to moderate, and male and female patients were appeared equally affected. MSSI scores ranged from 0 to 39 (mean 15; Table 1). Eleven patients were moderately and 12 patients were mildly affected (MSSI<20). None of patients were classified as having severe FD (MMSI>40). ENG was normal in all patients (amplitude, conduction velocity and distal motor latency), revealing no indication for a significant affection of the peripheral/autonomous nervous system (data not shown). Cardiac dysfunction with an ejection fraction ≤45% was detected in 6 patients (26%) who were all in a stable clinical state (New York Heart Association [NYHA] functional class I-II). All patients showed mild-to-moderate WMLs on conventional MRI (mean Fazekas score: 1.9; mean WML volumes: 2.97 mL). The symmetrical WM changes were most prominent in the parietal and frontal regions. No WMLs were found in the brainstem. Further details on imaging results are presented in Table 2.

**Table 1:** Demographic and clinical characteristics of patients and controls.

	Fabry Patients		Controls
	With CSA	Without CSA	
**n (women)**	5 (3); 22%	18 (8); 78%	44 (21)
Age [years]*	48±19	46±22	46±11
Total MSSI Score*	20.7±9.2	22.3±11.4	n.a.
**Cerebrovascular Events [n]**	0	0	0
**WML [n]**	5	18	0
**Neuropathic Pain [n]**	4 (80%)	14 (78%)	0
**Angiokeratoma [n]**	2 (40%)	8 (44%)	0
**Cardial Dysfuction (NYHA I-II) [n]**	2 (40%)	7 (39%)	0
Cardiomyopathy [n]	1 (20%)	4 (22%)	
Arrhythmia [n]	1 (20%)	3 (17%)	
Left Ventricular Hypertrophy [n]	0	2 (11%)	
LVEF<45% [n]	1 (20%)	5 (27%)	
Echocardiographic Data			n.a.
LVEDD [mm]*	47.9±9.2	50.4±8.3	
IVSD [mm]*	11.7±3.3	12.1±2.8	
EF [%]*	53.1±9.4	51.6±11.7	
**Renal Dysfuntion [n]**	3 (60%)	10 (56%)	0
Serum Creatinine [mg/dL]*	0.97±0.49	0.88±0.51	
eGFR [ml/min/1.73 m^2^]*	89.7 ±29.2	91.1±33.1	
Proteinuria>500 mg/24 h [n]	3 (60%)	11 (61%)	
ESS score*	13.5±8.1	9.4±9.2	3.7±5.8
BMI [kg/m^2^]*	24.2±8.2	26.2±9.8	28.1±11.2
Age at Diagnosis [years]*	43.2±19.0	42±15.3	n.a.
On ERT since [years]*	5.1±3.9	4.7±4.1	n.a.

Patients with CSA-CSR had a higher ESS score (P = 0.02). All other clinical and demographic data did not differ among both patient groups (P>0.05, two-sample t-tests).

CSA = Central Sleep Apnea; MSSI = Mainz Severity Score Index; WML = White Matter Lesions; LVEF = Left Ventricular Ejection Fraction; NYHA = New York Heart Association functional class; eGFR = estimated Glomerular Filtration Rate; ESS = Epworth Sleepiness Scale; ERT = Enzyme Replacement Therapy; LVEDD = Left Ventricular End-Diastolic Diameter; IVSD = Interventricular Septal Wall Thickness; EF = Ejection Fraction; BMI = Body Mass Index; *Mean±Standard Deviation.

**Table 2:** Means and Standard Deviations of the MR imaging analyses and polysomnographic parameter.

	FD patients without CSA	FD patients with CSA	P Values	Controls
	(n = 18)	(n = 5)		(n = 44)
**Brain Tissue Volumes**				
Normalized Brain Volume [mL]	1415.4±266.7	1431.0±236.7	0.344	1417.0±287.3
WM Volume [mL]	479.9±87.3	488.2±90.3	0.180	473.6±90.9
GM Volume [mL]	648.1±144.8	622.5±151.4	0.237	637.7±156.8
Brain/Intracranial Volume ×100 [%]	82.1±2.6	80.9±2.9	0.203	80.6±2.4
WML volume [mL]	2.86±1.44	3.14±1.03	0.149	n.a.
Fazekas Score	1.8±2.3	1.9±1.8	0.562	n.a.
**Polysomnographic Variables**				n.a.
Total Sleep Time [min]	362±122	328±90.8	0.682	
Sleep Efficiency [%]	78.9±12.8	62.7±10.3	0.015	
Stage 1 NREM [%]	8.4±7.8	8.5±11.0	0.662	
Stage 2 NREM [%]	73.4±13.1	75.1±15.2	0.347	
Stage 3 NREM [%]	4.0±7.6	3.9±5.6	0.214	
Stage REM [%]	14.1±10.8	12.4±10.9	0.389	
AHI [/h]	4.3±4.2	20.2±11.2	0.009	
oAHI [/h]	2.2±1.9	2.0±3.2	0.130	
cAHI [/h]	2.1±2.4	18.6±9.8	0.004	
Mean SpO_2_ [%]	96.7±4.3	94.3±4.0	0.076	
SpO_2_ Nadir [%]	90.2±6.0	77.8±7.7	0.009	
SpO_2_<90% [%]	6.1±11.1	14.2±12.5	0.026	
Arousal Index [%]	11.7±9.2	16.6±14.0	0.128	
PLMS Index [%]	11.4±18.0	12.7±17.2	0.403	
CSR [min]	n.a.	60.2±32.1		
CSR rel. [%]	n.a.	29.0±34.7		
				

There were no differences of brain tissue volumes between both patient groups or between FD patients and healthy controls. Values of lesion load did also not differ between both patient groups (two-sample t-tests).

Patients with CSA-CSR showed significantly decreased sleep efficiency, a higher AHI and cAHI, and a more severe oxygen desaturation compared to patients without CSA-CSR. Four patients with OSAS were found, two in both patient groups.

P-values in bold indicate significant differences between both patient groups.

Sleep Efficiency = total sleep time/total recording time ×100; Sleep stages: 1 = alpha rhythm is attenuated and replaced by low amplitude, mixed frequency for more than 50% of the epoch; 2 = presence of sleep spindles and K complexes on a background of low-amplitude, mixed frequency activity; 3 = slow wave activity in greater than 20% [2]. FD = Fabry Disease; CSA = Central Sleep Apnea; WM = White Matter; GM = Gray Matter; WML = White Matter Lesion; REM = Rapid Eye Movements; NREM = Non-REM; AHI = Apnea/Hypopnea Index; cAHI = central AHI; oAHI = obstructive AHI; SpO_2_ = Saturation of peripheral Oxygen; PLMS = Periodic Limb Movement; CRS = Cheyne-Stokes Respiration; CSR rel. = duration of CSR episodes/total duration of sleep.

No significant correlations were found between ESS scores or polysomnographic parameters and clinical (total MSSI scores and subscores, number of affected organs, body mass index) or demographic (age, gender, years of ERT, age at diagnosis) data. There were also no correlations between polysomnographic parameters (AHI, cAHI, oAHI, total and relative length of CSR episodes, mean SpO_2_, SpO_2_ nadir, SpO_2_<90%, sleep efficiency) and WML volumes, Fazekas scores, or brain tissue volumes.

Polysomnography revealed CSA-CSR in 5 of 23 FD patients (22%), and 2 patients (17%) showed obstructive sleep apnea syndrome or both (2 patient). Patients with CSA-CSR had a higher ESS score (13.5 vs. 9.4; P = 0.02).

There were no differences in all other clinical and demographic data among patients with and without CSA-CSR. Echocardiographic data (LVEDD: P = 0.228; IVSD: P = 0.172, and EF: P = 0.133), Fazekas scores (P = 0.562), as well as WML volumes (P = 0.149). Global brain volumes (P = 0.218) did also not differ between both patient groups.

**Figure 1:**
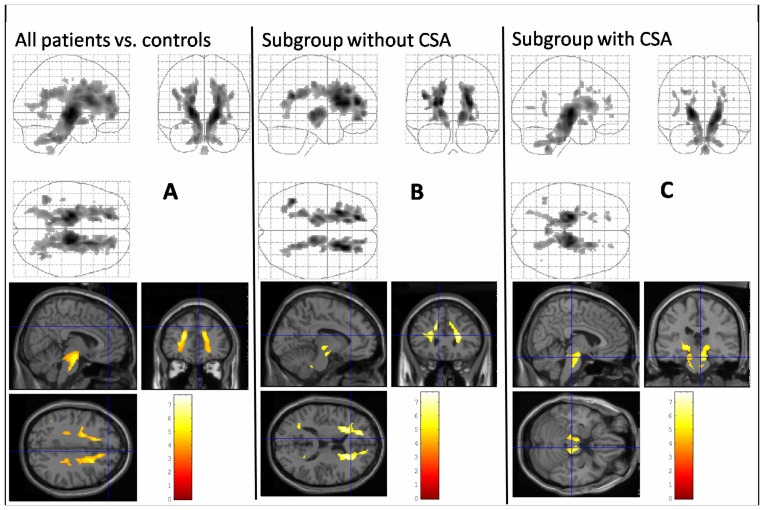
SPM "glass brain" representations (upper row) and statistical FA maps that were superimposed on a normalized T1-weighted template (lower row) showing clusters of microstructural damage of the FD patient group compared to healthy controls (ANCOVA, modelling age as a co-variate; p<0.001, corrected for multiple comparison; minimum of 50 contiguous voxels). Coloured bars represent t-values; display threshold is set at t value >3.16. A: FA values of the complete FD patients group (n = 23) were significantly reduced in WM areas covering widespread parts of the brain, indicating structural WM changes extending beyond the WM lesions that showed up on conventional MRI. B and C: Subgroup analyses of both patient groups (without (1B; n = 18) and with (1C; n = 5) CSA-CSR), relative to the healthy controls (n = 44). Clusters of FA changes in patients with CSA-CSR were most pronounced in the brainstem. By contrast, FD patients without CSA-CSR revealed more widespread FA decreases in supratentorial areas, but only subtle brainstem involvement. SPM  =  Statistical Parametric Mapping; FD = Fabry Disease; FA = Fractional Anisotropy; WM = White Matter; MRI = Magnetic Resonance Imaging; CSA-CSR = Central sleep apnea with Cheyne-Stokes respiration.

VBM analysis revealed no significant gray matter differences between FD patients and healthy controls, or between patients with and without CSA-CSR, whereas Voxel-based DTI-analysis revealed a widespread decline in FA in FD patients when compared to the healthy controls. The symmetrical WM changes were most prominent in the frontal lobes, the midbrain, and in the brainstem (Figure 1A). In general, the clusters of FA reductions significantly exceeded the WM hyperintensities that were seen on conventional MRI.

When calculated as a separate group, pattern of FA changes in the patients with CSA-CSR were most prominent in the brainstem, relative to the healthy controls. By contrast, FD patients without CSA-CSR revealed more widespread FA decreases in supratentorial areas, but only subtle brainstem involvement (Figures 1B and 1C).

To control for the influence of cardiac impairment on the MRI findings, we further calculated voxel-based DTI and VBM analyses by splitting our study group in those with (NYHA I-II; n = 9, 2 with CSA, see Table 1) and without cardiac impairment (n = 14, 3 with CSA). Both analyses revealed no significant differences in gray matter volumes or WM damage between both groups (p>0.05). There were also no differences in polysomnographic parameters between patients with and without cardiac involvement (p>0.05)

**Figure 2:**
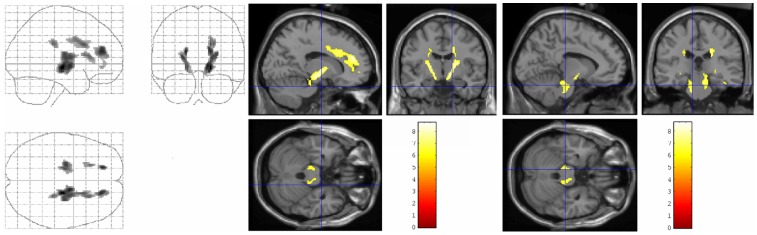
Brain regions in which microstructural lesions were associated with the severity of CSR in patients with CSA. SPM “glass brain” presentation (left) and statistical FA maps that were superimposed on a normalized T1-weigthed template (middle and right) showing symmetric clusters of correlation between relative CSR length and decreased FA values in the upper brainstem and in frontal WM as well as in connecting fibres between both areas (p<0.001, minimum of 50 continuous voxels; corrected for multiple comparisons). Coloured bars represent t-values; display threshold is set at t-value >3.71. CSR = Cheyne-Stokes Respiration; CSA = Central Sleep Apnea; SPM = Statistical Parametric Mapping; FA = Fractional Anisotropy.

Voxelwise regression analysis of FA values and relative length of CSR episodes revealed significant clusters in the brainstem and in frontal WM, as well as in connecting fibres between both domains indicating negative correlations (i.e., more pronounced WM damage in these areas predicted more severe CSR) (Figure 2). Interestingly, the clusters were located in similar areas as the above mentioned FA changes, which were found when the CSA-CSR group was compared to the healthy controls. No positive correlations were found. Interestingly, there were also no correlations between FA values and any other polysomnographic parameter or clinical involvement (quantified by MSSI). In particular, we found no correlation between FA changes and cardiac involvement, quantified by echocardiography. No voxels with significant correlations between polysomnographic parameters and gray matter values were found.

## Discussion

There are two important novel observations that have emerged from this detailed study of CSA-CSR in patients at risk of cerebral WMLs. Firstly, more than one in five FD patients with WMLs on conventional MRI suffer from CSA-CSR. Secondly, microstructural damage of neural networks within the brainstem could be the neuroanatomical correlate of CSA-CSR. Moreover, the severity of central sleep-disordered breathing in our FD patients correlates with the severity of structural brainstem damage detected by DTI. Thus, these microstructural DTI changes within the brainstem seem to be an anatomical marker of disease severity in CSA-CSR.

Our findings of an involvement of brainstem neural networks is in agreement with observations by other groups that contributed CSA-CSR to damage or alteration (either direct or indirect) in central respiratory control centres within the brainstem [17], [50], [51]. However, most MRI studies failed to show consistent data of structural cerebral damage in these patients [3]. One reason might be that only very few studies with a small number of patients were conducted, mainly using conventional MRI techniques. These diagnostic tools are known to be insensitive in uncovering the subtle microstructural but functional relevant lesions within the brainstem [52]–[54]. In fact, the hypothesis was supported by several case series and recent case reports that have impressively shown a primary association between brainstem damage and CSA-CSR [6], [8]–[12], [55], [56]. CSA-CSR was often the first and only neurological symptom of various diseases involving the brainstem. Some authors postulated that the brainstem respiratory network contains neurons critical for respiratory rhythmogenesis receiving inputs from peripheral and central chemoreceptors sensitive to levels of carbon dioxide and oxygen. Taking these results into account, the patterns of DTI changes that were found in the present study are most likely the structural correlate of either primary or secondary changes of central respiratory control centers in CSA-CSR patients.

Patients with FD commonly have cardiac involvement, in particular left ventricular hypertrophy [57]. It is tempting to speculate that mainly the cardiac involvement caused the dysregulation of nocturnal breathing pattern resulting in CSA-CSR in the here described patients. However, although 6 of 23 patients of the present study showed a mild Fabry-typical cardiac involvement (EF≤45%, only one with an EF<30%), we found no association between cardiac parameters and the severity of CSA-CSR. Moreover, FD patients without CSA-CSR even showed a more (but not significant) severe cardiac involvement. This finding is backed by other studies revealing that the ventricular dysfunction is not directly associated with the severity of the CSR [58]. We also found no differences in gray matter or WM damage analyses when splitting the patients and comparing those with and without cardiac involvement. Both groups (with and without cardiac impairment) did also not differ in polysomnographic parameters.

However, epidemiological data have shown a high prevalence of central sleep apnea in patient groups with heart involvement. Depending on the NYHA classification and the diagnostic methods, it varies around 40 to 60% [59] or even more [60]. The occurrence of Cheyne-Stokes respiration, which is a marker of the severity and prognosis of heart failure, is less frequent and is observed in about 15% [61] to 30% [62] of stable patients with cardiac impairment. Since 26% of our patients showed a mild to moderate cardiac impairment (EF<45%) with clinical stability, one would expect CSA-CSR in about 3.9 to 7.8% or only one to two of our patients.

Taken together, we can not entirely exclude that the microstructural changes in the brainstem are typical for Fabry disease but not for central sleep apnea. Although we also could not exclude a basic cardiac influence on CSA-CSR in our patients, we believe that we have thoroughly examined this cardiac impact. Thus, it is unlikely that the slight left ventricular systolic dysfunction in a small part of FD patients alone led to central sleep apneas. However, the combination of cardiac dysfunction and structural cerebral damage might well lead to a predisposition of developing CSA-CSR and could explain the high incidence(>20%) in our patient group. Further studies are necessary to address these issues. We particularly suggest investigating patients with heart failure by using the same methodological approaches. However, even then it still remains to be established whether the MRI alterations are a cause or a consequence of CSA.

Beside the association between brainstem changes and CSR, we also found supratentorial lesions that were associated with CSA-CSR. Screening of the literature revealed that numerous supratentorial areas have been reported to interact with brainstem neurons like the reticular formation [63]. The result of the present study points to an influence of frontal areas that are particularly connected with neurons of the reticular formation. Our finding of an association between frontal- and brainstem lesions and increasing length of CSR episodes is suggestive of disturbance or even more a damage of WM projections in frontal-brainstem pathways leading to central breathing dysregulation. These findings are supported by a study using functional MRI [64] and recent studies in stroke patients reporting patients with ischemic lesions in frontal areas to be particular affected by CSA-CSR [65].

DTI has a unique capability to detect subtle degeneration within the *white matter*. Although high-resolution volumetric measurements revealed no correlations between clinical data and gray matter changes, neurons of respiratory control centers within the brainstem might be primarily affected and the FA reductions are most likely atrophic WM changes or Wallerian degeneration secondary to a gray matter reduction. Since neurons of the reticular formation consist of more than 100 small neural networks, a minor structural disorganisation of aggregated neurons might not be disruptive enough to cause significant alterations on Voxel-based analysis of gray matter, but the subtle changes of neural tracts within the networks could be picked up by DTI. Thus, DTI seems to be much more sensitive than VBM to detect subtle degenerative changes of respiratory control centers within the brainstem. These findings were in accordance with other studies showing that DTI changes correlate with clinical symptoms, histopathological changes, and functional deficits in early stages of vascular and degenerative brain damage [23]–[25], [66]. Furthermore, DTI has a proven sensitivity to detect subtle structural brain changes even in primary cortical degenerative disease, and FA was shown to be the most sensitive MR-imaging correlate of neurodegeneration [23], [67], [68].

A restriction of voxel-based DTI analyses is the limited spatial resolution. However, we used a high-resolution sequence with small voxel size (1.8×1.8×3.6 mm) and no gaps at 3 Tesla. Additionally, the extent of neural clusters of, for example, the reticular formation reported in the literature varies significantly. Thus, it is unlikely that even when using a MR-technique with higher spatial resolution, a more precise localization of those loose and intricate cell aggregates in the brainstem would be detectable and would lead to more reliable results.

## Conclusions

This is the first study applying DTI in patients with CSA-CSR. We conclude that alterations of the nerve fibers associated with brainstem neurons and connection fibers from frontal regions seem to be an anatomical marker for disease severity in CSA-CSR. While it remains to be established whether DTI alterations are a cause or a consequence of CSR, or both, they could constitute an important step towards understanding models of the possible underlying pathophysiological mechanisms in CSR.

We conclude that DTI is more sensitive and specific than conventional structural MRI and other advanced MR analyses tools (VBM, automated brain tissue volumetry) in demonstrating brain abnormalities in patients with CSA-CSR. From a clinical point of view, we may suggest DTI as a new sensitive tool to detect structural brain changes associated with central sleep apnea. Future studies on patients with other pathways to central sleep apnea (e.g. heart failure) should be assessed to confirm these findings. If so, studies with larger samples should evaluate the ability of therapeutic interventions to change these structural cerebral changes in individuals with CSA-CSR. Since patients with CSA-CSR differently respond to ventilatory therapies (e.g. CPAP or autoservo ventilation), it might be interesting to see if these patients show a specific pattern of microstructural cerebral changes.
